# Dental restoration operative time and analysis of the internal gap of conventional resins (Incremental Technique) vs. Bulk Fill (Single-Increment Technique): *In vitro* study

**DOI:** 10.4317/jced.60717

**Published:** 2023-08-01

**Authors:** Priscilla Medina-Sotomayor, Gabriela Ortega, José Aguilar, Paola Ordóñez, Michelle Rojas, Roxana Vásquez

**Affiliations:** 1 Universidad Católica de Cuenca, Carrera de Odontología, Campus Universitario Azogues, Ecuador

## Abstract

**Background:**

To determine the operative time and the internal restoration gap in the restoration–tooth interface in the cavity floor using an incremental technique for conventional resins and the single-increment technique for the bulk fill resin.

**Material and Methods:**

In this *in vitro* experimental study, the internal gaps of the restoration–tooth interfaces in the cavity floors of two conventional resins and two bulk fill resins were microscopically analyzed, and the restoration times of the single-increment technique and the incremental technique were determined.

**Results:**

Bulk fill resins had smaller internal gap (63.31 µm) than conventional resins (333.14 µm). Regarding the restoration time, the single-increment technique obtained the best results in operative time (3.52 minutes), with significant differences relative to the incremental technique.

**Conclusions:**

The Tetric N-Ceram bulk fill resin presented better performance than conventional resins relative to the internal gap of the restoration–tooth interface in the cavity floor. In addition, the single-increment technique presented a short clinical restoration time.

** Key words:**Composite, internal gap, restoration technique.

## Introduction

Restorative dentistry is constantly evolving, as it requires materials that provide quality to this type of procedure due to the various causes that lead to failed restorations. For this reason, clinical practice is focused on restoring the integrity of the dental organ with the use of adhesive materials. Certain directives, such as composite resin, have been on the market since the 1950s and do not require the removal of healthy tissue for their retention, obtaining excellent long-term results.

The conventional resin has the great disadvantage of contracting during the polymerization process because the tension of the material exceeds the bonding force due to the low percentage of inorganic load in its composition (1-3). This phenomenon creates an internal gap that causes microfiltration between the restoration–tooth interface, generating microorganisms that are responsible for secondary carious lesions, pulp damage and failure in the restoration (4-11).

Bulk fill resins are materials that have been implemented in the market to meet the needs and improve the properties of conventional resins and restoration techniques. The resins are characterized by their low polymerization contraction due to their high percentage of inorganic load in their composition (quartz, zirconite or silicate) and photoinitiators with translucent characteristics that allow the passage of light into the restorations; these characteristics improve the contraction of polymerization and allow the passage of light to a great depth, improving restoration techniques (5,10,12).

Due to the uncomfortable clinical process of dental restoration, it is recently a matter of minimizing the operative time of restoration through the practice of different techniques (13). The incremental technique used in restorations with conventional composite resin promises reduced shrinkage and increased working time because it consists of applying small increments of resin within the cavity preparation accompanied by light curing at intervals, which can generate gaps between increments (6,13,14). However, bulk fill resin allows the use of the single-increment technique, improving the operative time and facilitating the restoration (6,15).

The objective of this *in vitro* study is to determine the operative time and the internal restoration gap at the restoration–tooth interface in the cavity floor using the incremental technique for conventional resins and the single-increment technique for the resin.

## Material and Methods

-Sample calculation and selection

In the present *in vitro* study, 40 third molars obtained from the Dental Teaching Clinic (surgery area) of the Universidad Católica de Cuenca, Azogues were analyzed. The sample size was 10 teeth for each composite resin group; the sample size was determined prior to a pilot study.

As inclusion criteria, upper or lower third molars were used with the same Nolla stage without carious lesions, fractures, or restorations and with a complete crown. Only teeth with the possibility of fracturing or cracking were excluded during the experimental phase.

The composite resins Filtek Bulk Fill Posterior Restorative (FB) and Tetric N-Ceram Bulk Fill (TB) were analyzed for the restorations with the single-increment technique, and the Filtek Z250 XT Universal Restaurative (FZ) and Tetric N-Ceram (TC) were analyzed for the restorations with the technique. The incremental and compositional characteristics are indicated in Table 1.

**Table 1:**
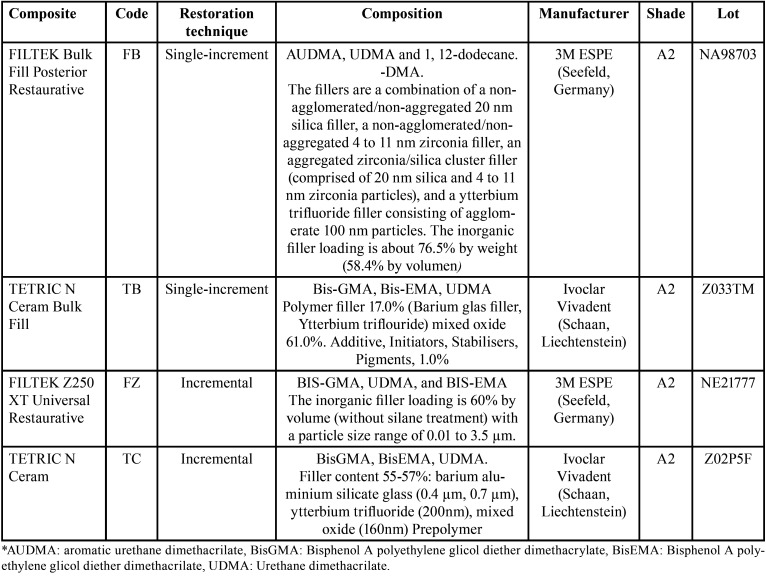
Composite descriptions, manufacturers and composition.

A randomization (www.random.org) of the teeth was carried out to define the technique and resin to be used, and the information was managed by one researcher.

-Tooth preparation

The teeth were cleaned with a prophylactic brush and pumice stone, and the soft tissue remains were removed with a curette. The apex was sealed with glass ionomer (GC Gold Label 9, FUJI, Tokyo, Japan) and then kept in physiological serum (NaCl 0.09%) at 6 °C, changing it twice a week until the experimental part began. Each tooth was placed in individual sockets to achieve stability at the time of cavity preparation and subsequent restoration.

We proceeded to make standardized class I cavity preparations of 4x4x4 mm in width, length and depth, ensuring measurements with the use of the periodontal probe. The cavity opening was made with a round bur, and the samples were prepared with a straight-tipped tapered bur using rubber stops as guides.

-Cavity conditioning and restorations

The restorations were performed by a single operator controlling the operative time from the beginning of each restoration. The restorations were performed according to the specifications of each manufacturer, and a PhotoLight Max light curing lamp (Gnatus, Riberão Preto-SP, Brazil) was used with a light-emitting diode (LED) light power of 1200 mW/cm2 with continuous light as follows:

For one-piece technique restorations (FB–TB), the following steps were conducted:

• Acid selective conditioning the tooth structure, first conditioning the enamel and then conditioning the dentin, for 15 s with Scotchbond Universal Etchant (3M ESPE, Seefeld, Germany. LOT NA98703)

• Washing the cavity with running water for 15 seconds and then drying the enamel while leaving the cavity/dentin wet

• Placement of 2% chlorhexidine for 10 seconds and cavity drying with cotton

• Adper Single Bond 2 adhesive (3M ESPE, Seefeld, Germany. LOT: NE63329) was applied on FB resin and N-Bond (Ivoclar Vivadent, Schaan, Liechtenstein. LOT: Z033TM) was applied on TB resin as the first layers for both the enamel and dentin by rubbing the dentin for 10 seconds

• Unifying the first layer with air application

• Placement of the second layer of adhesive and light curing for 10 seconds

• Restoration with polymerization for 40 seconds

For restorations with the incremental technique (FZ–TC), the following steps were performed:

• Acid selective conditioning the tooth structure, first conditioning the enamel and then conditioning the dentin, for 15 seconds with Scotchbond Universal Etchant (3M ESPE, Seefeld, Germany. LOT: 7601007)

• Washing the cavity with running water for 15 seconds and then drying the enamel while keeping the cavity/dentin wet

• Placement of 2% chlorhexidine for 10 seconds and cavity drying with cotton

• Adper Single Bond 2 adhesive (3M ESPE, Seefeld, Germany. LOT: NE63329) was applied on FZ resin and N-Bond (Ivoclar Vivadent, Schaan, Liechtenstein. LOT: Z033TM) was applied on TC resin as the first layers for both the enamel and dentin by rubbing dentin for 10 seconds

• Unifying the first layer with air application

• Placement of the second layer of adhesive and conducting light curing for 10 seconds

• Restoration curing each layer for 15 seconds and performing final curing for 40 seconds

-Thermocycling, preparation and immersion of teeth in dye

Subsequently, thermocycling was carried out with 10,000 cycles, representing 1 year of use of the material in oral conditions as follows: (16) the teeth were immersed in water baths at 35 °C for 28 seconds, 15 °C for 2 seconds, 35 °C for 28 seconds and finally 45 °C for 2 seconds. This procedure was repeated 50 times for 3.5 days.

-Sample sectioning and observation

The pieces were washed and sectioned mesiodistally with a handpiece and a diamond disc with constant irrigation. Finally, the teeth were immersed in 0.1% methylene blue for 48 hours. After this time, the sealing was evaluated marginally at the mesial, distal and floor levels of the cavity using a Dino-Lite Premier AM4113T 1.3MP optical microscope (Dino-Lite Digital Microscope, Taiwan) and subsequent analysis with DinoCapture 2.0 software (Dino-Lite Premier, Taiwan). This analysis was performed by a single operator blinded to the material analyzed (JA).

-Statistical analysis

For the statistical analysis, the Statistical Package for Social Sciences (SPSS) v.25 program (IBM, Endicott NY, USA) was used. Descriptive data were obtained, and through Student’s t test, restoration techniques and operative time were related. With the Mann‒Whitney U test, the means of the internal restoration gap were compared according to the type of resin. Significant values were established at *p* <0.05.

## Results

**Figure 1:**
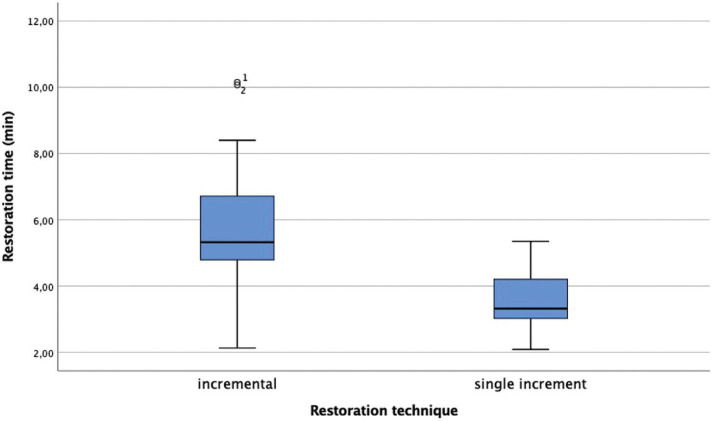
Restoration time (min) according the restoration technique.

The descriptive analysis of the time according to the restoration technique is indicated in Table 2. The single-increment technique used with the Bulk fill resins obtains an average of 3.52 minutes with significant differences relative to the incremental technique (*p* <0.05) (Fig. 1).

**Table 2:**
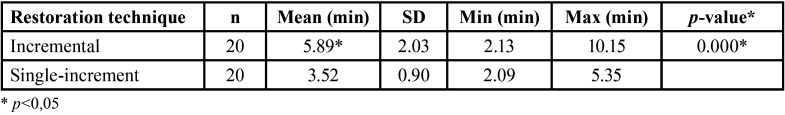
Restoration time according the restoration technique descriptive analysis: mean value, standard deviation, mínimum and máximum value. (µm) Statistical analysis: T test.

**Figure 2:**
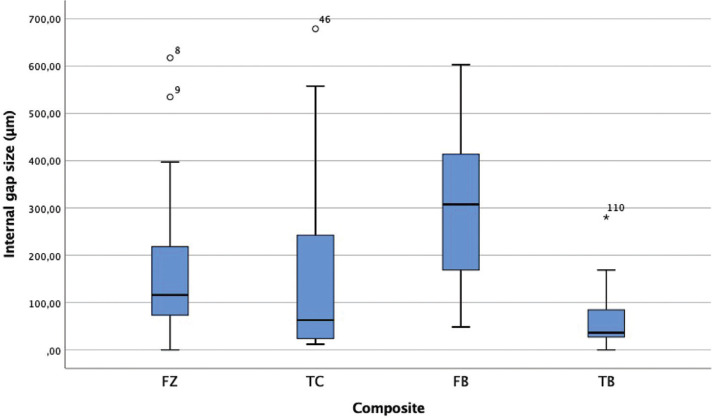
Dental restoration internal gap size (µm). * FZ: FILTEK Z250 XT Universal Restaurative; TC: TETRIC N Ceram; FB: FILTEK Bulk Fill Posterior Restaurative; TB: TETRIC N Ceram Bulk Fill.

**Figure 3:**
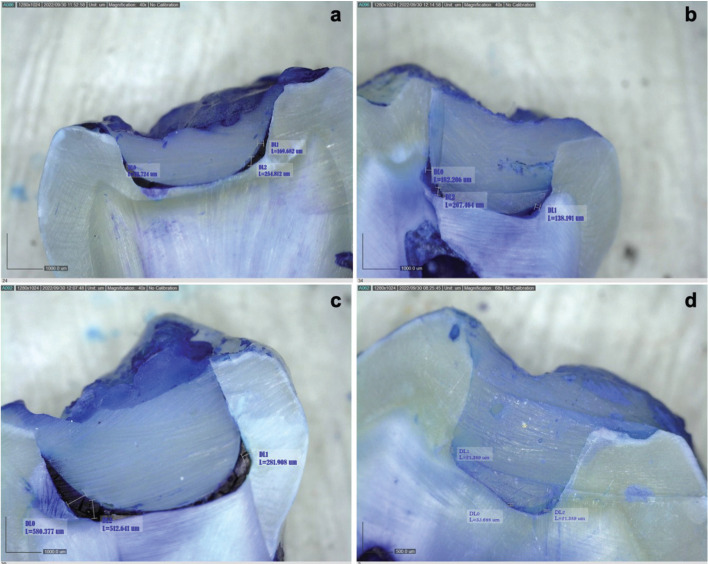
Dental restoration internal gap size (µm) microscopical view. * a: FZ (FILTEK Z250 XT Universal Restaurative); b: TC (TETRIC N Ceram FB); c: FB (FILTEK Bulk Fill Posterior Restaurative); d: TB (TETRIC N Ceram Bulk Fill).

Figures 2 and 3 show the size of the internal gap of the restoration of the four resins considered for the present study. Table 3 shows very different behaviors between the two bulk fill resins (*p* <0.05), with the TB resin having the smallest internal gap size at an average value of 63.31 µm; the FB resin have the highest value of all the resins, with an average value of 313.14 µm. The two conventional resins (FZ and TC) present very similar average values (*p*> 0.05).

**Table 3:**
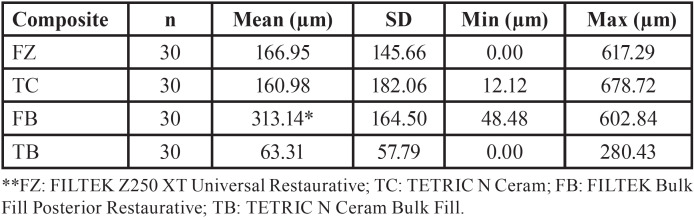
Dental restoration internal gap size descriptive analysis (mesial, distal and floor levels of the cavity of each sample): mean value, standard deviation, mínimum and máximum value, (µm).

According to a comparison between groups (Table 4), the FB and FZ resins of the same commercial brand present significant differences between them (*p* <0.05).

**Table 4:**
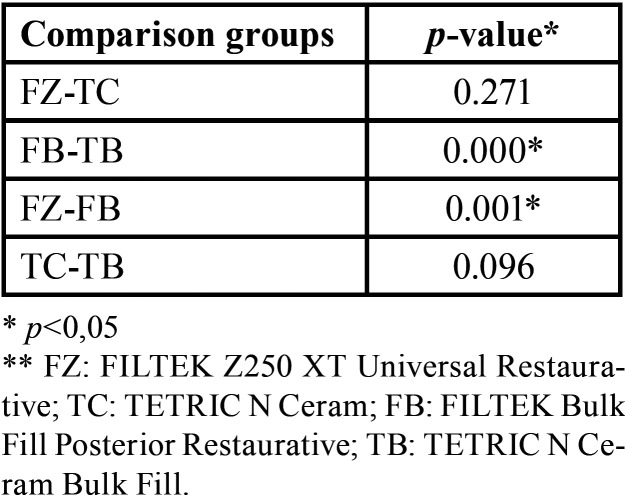
Dental restoration internal gap size statistical analysis: Mann-Whitney.

Finally, relative to the size of the internal gap according to the location, the TC, FB and TB resins present high values at the restoration floor level, while the FZ resin presents high values at the mesial level (Table 5).

**Table 5:**
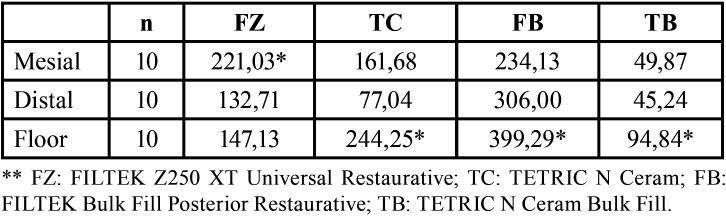
Dental restoration internal gap size mean (µm).

## Discussion

In the present study, the internal gap formed between the restoration–tooth interface and the restoration operative time between bulk fill resins and conventional resins are observed; this analysis is crucial due to the importance of improving the clinical experience of a patient during restorations.

Composite resin is the most commonly used resin in direct adhesive restorations due to its aesthetic characteristics and mechanical properties (4,17,18). One of the great advantages of this material is that it does not require the removal of healthy tissue for its retention and that it has good mechanical behavior in the posterior sector (4,18,19). However, the disadvantages include the formation of secondary caries, fracture of the restoration, microleakage and marginal discoloration, which are all caused by polymerization shrinkage (4).

This contraction is responsible for the presence of marginal leaks, a high coefficient of thermal expansion and the absorption of water, impoverishing the internal adaptation and forming gaps between the restoration material and the dental structure, which allows the filtration of fluids, thus affecting long-term retention; however, the success of restorations depends on shrinkage stress and various other factors that impoverish the marginal seal (18,20,21).

The results of this study show that bulk fill resins present smaller internal gaps than conventional resins, even from the same commercial company, in cavitary preparations with 4-mm depth; the Tetric N-Ceram Bulk Fill presents the best results (63.31 µm), with other studies that corroborate these findings (15,17).

There are several factors to consider that can generate failures in the restoration–tooth interface, forming an internal gap that causes microfiltration that is responsible for postoperative sensitivity and secondary caries (21,22), such as cavitary preparation, restoration, adhesion, light irradiation, exposure time and the properties of the composite resin.

Bulk fill composite resins have small inorganic particle sizes, increasing the depth of light curing and allowing the passage of light in a better manner, reducing volumetric contraction and internal gap formation (23-27).

Regarding the restoration technique, it is important to indicate that the restoration time is measured from the moment the resin is introduced into the cavity preparation; this indication avoids confusion with the time necessary for the clinical preparation of the patient and for the placement of the adhesive system in each restoration. It is possible to determine that the single-increment technique used in Bulk Fill resins compared to the incremental technique improves clinical restoration times with significant differences, avoiding the gaps that the interfaces between layers may present when the incremental technique is conducted, improving marginal sealing. Notably, class I cavity preparations are used to improve Factor C (conFiguration factor), which can strongly affect the polymerization stress of the composite resin (28).

The FZ resin presents the greatest restoration–tooth interface with a value of 166.95 µm. The depth of the cavity preparation may affect the polymerization stress forming the internal gap because, despite using the incremental technique, the depth exceeds the specifications of conventional composite resins (1,5,6).

A two-step adhesive is used because studies indicate that etch-and-wash adhesives generate a thick hybrid layer with long, wide and dense resin layers; at this point, the filtration in dentin is not significant because the dentin tubules are sealed after the application of the adhesive, thus avoiding microfiltration because greater control is maintained in the time of application of the phosphoric acid and adhesive. In addition, chlorhexidine is used, which has been shown to improve the resistance of the hybrid layer (29). In this manner, it is possible to control errors in the adhesive sealing, improving the clinical restoration protocol (30,31).

It is important to remember that polymerization contraction affects the entire restoration–tooth interface. At this level, adding a poor-quality material can become more damaging to the dental structure.

## Conclusions

By considering the limitations of the present *in vitro* study, it can be concluded that bulk fill resins present smaller internal gaps between restoration–tooth interfaces than conventional resins in 4 mm deep class I cavity preparations. In addition, single-increment restoration technique improves clinical restoration times relative to the oblique incremental technique.
